# Visual attention at the tip of the tongue

**DOI:** 10.1068/i0697sas

**Published:** 2015-01-06

**Authors:** Michael Barnett-Cowan, Matin Soeizi, Joseph F. X. DeSouza

**Affiliations:** Department of Kinesiology, University of Waterloo, Waterloo, Ontario, Canada; e-mail: mbc@uwaterloo.ca; Department of Psychology, Centre for Vision Research, York University, Toronto, Ontario, Canada; e-mail: matinsoeizi@gmail.com; Department of Psychology, Centre for Vision Research, York University, Toronto, Ontario, Canada; e-mail: desouza@yorku.ca

**Keywords:** attention, body, embodied cognition, somatosensory, tongue, visual search

## Abstract

The brain shifts attention by selectively modulating sensory information about relevant environmental features. It has been shown that eye, head, trunk and limb position can bias spatial attention. This leads to the interesting question: Does the brain only recruit bodily information that is explicitly related to orienting behaviour to direct attention, or more generally? We tested whether tongue position, which does not explicitly functionally relate to orienting behaviour, biases attention in a visual search task. Thirty-six participants completed three visual search trial blocks of increased difficulty each consisting of three tongue positions for 50 trials. Response times and error rates were used to assess whether tongue position modulates visual attention. Results show that sensorimotor information from the tongue modulates attention in a difficult visual search task: faster responses to visual search targets presented ipsilateral with the tongue; slower responses when contralateral. In line with cognition being generally embodied, the tongue plays a surprising role in directing attention.

To efficiently process sensory information, the brain shifts attention by selectively modulating sensory information about relevant features in the environment (Itti, Rees, & Tsotsos, 2005). The mere displacement of the eyes (Craighero, Nascimben, & Fadiga., 2004), head (Schindler & Kerkhoff, 1997), trunk (Grubb, Reed, Bate, Garza, & Roberts, 2008) or limb (Driver & Grossenbacher, 1996) can bias a participant's attention in space. Whether the brain only recruits bodily information that is explicitly related to orienting behaviour to direct attention, or in a general manner is still debated. For example, the position of the eyes, head, limbs and trunk all functionally relate to orienting behaviour when searching visual targets which may confound embodied cognition theory (Wilson, 2002). However, if a body part not explicitly associated with orienting behaviour (e.g. the tongue) biases attention, this would provide compelling evidence of bodily information being generally recruited to direct attention.

Why should the tongue modulate attention? Compared with other body parts, the tongue is represented by large primary motor and sensory cortical areas (Penfield & Rasmussen, 1950; Figure 1a). This corresponds with the tongue having the highest nerve fibre to muscle fibre ratio following the eye muscles (Silverman, 1961), and the tip of the tongue having greater tactile sensitivity than the fingertip (Ringel, 1970). Thus, although the tongue is associated as the primary organ of taste, it is used to manipulate items (usually food), and is involved in speech production through phonetic articulation, sensorimotor information from the tongue and/or simply tactile information from the tongue-in-cheek may be recruited by attentional mechanisms known to recruit position information from other body parts to facilitate tasks such as visual search. If, however, only bodily information explicitly related to orienting behaviour is recruited for visual search, then we would expect no effect of tongue position on visual search. To assess these competing hypotheses, participants in our study held their tongue in different positions (Figure 1b) while performing a visual search task (Figure 1c).

**Figure 1.**
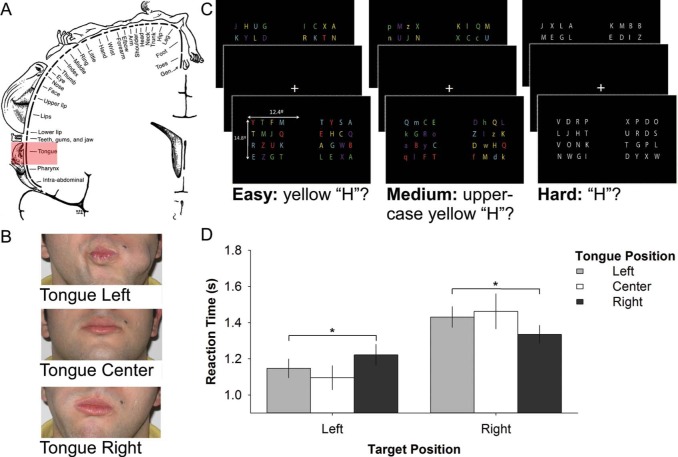
(a) The tongue is represented by a large cortical area shown highlighted here in Penfield's sensory homunculus (Modified from Penfield, & Rasmussen, 1950). (b) Tongue positions used in testing. (c) Visual search task of varying difficulty. (d) Response times to left and right targets of hard difficulty for each tongue position. *: *p* < 0.05. Error bars are S.E.M.

Fifty-one participants with normal vision gave informed and written consent to participate in the experiment, which was approved by York University's ethics committee, which complies with the Declaration of Helsinki. Complete data were not collected from 15 participants due to the inability to perform the task (4), withdrawal (6), and technical difficulties (5). Data from the remaining 36 participants (18 female; mean age 20.2 years, s.d. 3.03) were analyzed.

Participants completed three visual search trial blocks each consisting of three tongue position conditions of 50 trials each. Block and condition order were randomized across participants. Throughout a given condition, participants held their tongue in either their left cheek, their right cheek, or the center of their mouth (Figure 1b). A mirror was placed in front of participants for the experimenter to monitor tongue position ensuring that the tongue was held in the desired position throughout all trials. Visual stimuli were presented on a 48 cm (diagonal) ViewSonic colour LCD monitor (1280 × 1024; Presentation v14.9 software). Stimuli were viewed binocularly (50 cm distance) in a semi-darkened lab. A chin rest minimized head movements.

In each block, a target letter was present in 50% of trials. Trials were initiated with a central fixation cross (1s) followed by the stimulus (2s). Participants fixated a central cross between trials. Stimuli were composed of two arrays of 16 randomized letters (4 × 4 grid) on the left and right side of the peripheral visual field. The maximum horizontal and vertical visual angles from fixation were ± 12.4° and ± 7.1°, respectively. Participants looked for a target letter that differed slightly in each of the three blocks, were told to fixate on the target, and respond via keyboard press when they found the target (left and right Ctrl keys for left or right targets, respectively). No response was required for trials in which the target letter was not present.

The three visual search blocks were similar but had different levels of difficulty. Letters in the easy task were presented in different colours (Figure 1c, left). Here, the target was a yellow letter “H.” The moderate task consisted of upper and lower case letters of different colours and the target letter was a yellow letter “H” (Figure 1c, center). The hard task consisted of white letters and the target was the letter “H” (Figure 1c, right). As correct responses to target letters involved colour recognition, all colours used in the stimuli were shown to participants prior to testing where the target colour was indicated by the experimenter to control for colour blindness. Response times and error rates were used as dependent measures to determine whether tongue position modulates visual attention. These were submitted to separate repeated measures ANOVA (3 × difficulty; 3 × tongue position; 2 × target position). The Greenhouse–Geisser correction was used to correct for deviations from sphericity.

The results show that responses slowed with task difficulty *F* (1.6, 54.388) = 42.4, *p* < 0.001, that responses were faster for targets on the left compared to the right *F* (1,34) = 28.4, *p* < 0.001, and a significant interaction between tongue and target positions *F* (1.897, 64.482) = 4.0, *p* = 0.025. This interaction was only significant for the hard difficulty level *F* (1.602, 54.463) = 3.6, *p* = 0.042, such that visual search responses are faster to targets presented ipsilateral with the tongue; slower responses to targets contralateral of the tongue when attentional demands are high (Figure 1d). Differences between error rates did not reach significance.

While the position of the eyes, head, limbs and trunk all functionally relate to orienting behaviour when searching visual targets, the tongue is not explicitly associated with visual search. Our results showing that tongue position modulates visual search provide compelling evidence that the brain recruits bodily information in a general manner to direct attention. This general recruitment of sensory and motor processes, whose primary function is to enable us to interact with the environment, is in line with theories of embodied cognition (Wilson, 2002). As the tongue normally adopts a central position, it might be recruited to reinforce head position. However, as we show, this assumption comes with a cost when the tongue is offset. An alternative interpretation is that tactile information alone, which biases visual spatial attention (Spence, Nicholls, Gillespie, & Driver, 1998), from the tongue-in-cheek could be sufficient to bias attention without requiring engagement of sensorimotor information or body representation. We suggest that the tongue's surprising role in modulating attention should be considered in attention research, vision science and in developing tongue-operated interfaces for people with severe disabilities (Huo & Ghovanloo, 2010).
